# A ‘new’ NICE for health and social care

**DOI:** 10.1308/003588412X13373405386817

**Published:** 2012-10

**Authors:** Gillian Leng

**Affiliations:** Chair, Interventional Procedures Advisory Committee; Chair, Medical Technologies Advisory Committee; National Institute for Health and Clinical Excellence, MidCity Place, 71 High Holborn, London WC1V 6NA. www.nice.org.uk

The role NICE plays in advising the NHS on how its professionals can deliver high-quality healthcare has continually grown since its establishment twelve years ago. It has diversified significantly from its original remit in 1999 to evaluate the cost-effectiveness of licensed medicines and now advises on most aspects of healthcare, including surgical procedures as well as public health.

This expansion will continue next April when NICE will become responsible for developing guidance and quality standards for adult and children’s social care services in England. This additional work will be accompanied by some important changes to NICE’s organisational status but NICE’s key guidance outputs for clinical care and public health will continue as before.

## What are quality standards?

Quality standards are a set of specific and measurable statements (typically ten statements per standard), which define what high-quality and cost-effective care should look like for the treatment and prevention of different diseases and conditions. They are based on existing high-quality guidance (such as NICE guidance and other NICE-accredited sources) and address three dimensions of quality:

1. effectiveness
2. patient safety
3. patient experience

Quality standards are designed to be aspirational but entirely achievable statements and allow organisations to identify priorities for improvement and achieve excellence. Professionals use them in clinical audit and governance reports, and they will also underpin the indicators in the Quality and Outcomes Framework (the QOF). Commissioners will use them in setting contracts and to inform Commissioning for Quality and Innovation payments.

The standards are a crucial part of the government’s vision to have an NHS focused on delivering the best possible outcomes for patients. This was first detailed in the government’s white paper: *Equity and excellence: Liberating the NHS* (2010), in which the government set out a target for NICE to develop 150 quality standards over the next five years (this has since increased to 180). This vision is supported in the Health and Social Care Act 2012, which also states that NICE will support the NHS Commissioning Board to develop the Commissioning Outcomes Framework (COF). Quality standards will be used to inform a set of COF indicators, similar to the QOF, which will be used as part of a set of measures to hold clinical commissioning groups to account for the health outcomes and quality of care in their local populations.

Between June 2010 and August 2012, NICE published 21 quality standards for healthcare professionals and commissioners. These have covered topics such as chronic heart failure; hip fracture; and breast, colorectal, lung and ovarian cancers. In addition to these, last March the government referred the remaining healthcare quality standard topics to NICE, including ones on varicose veins, hernia, surgical site infection and sepsis, perioperative care, peripheral vascular disease, and medicines adherence. The full library of NICE’s 180 healthcare quality standards are available to view online at: www.nice.org.uk/qualitystandards.

It is encouraging that the government recognises the value quality standards bring to healthcare and their potential for improving the quality of care within England’s social care sector.

## Health and Social Care Act 2012

In March, the government’s Health and Social Care Act 2012 was passed into law. It outlines plans for a ‘new’ NICE to be established, which will be responsible for developing quality standards for NHS, public health and social care services in England. Social care is a significant addition to NICE’s remit and follows NICE’s track record in developing robust evidence-based guidance in healthcare and public health and its reputation for involving a wide range of stakeholders in its work.

As part of this change, NICE will transfer from being a special health authority of the NHS and be re-established as a non-departmental public body, called the National Institute for Health and Care Excellence (still referred to as NICE). NICE’s clinical and public health guidance programmes, including NICE’s interventional procedures guidance (commonly featured in the *Annals*) will continue and will not be affected by this transition.

## Work so far

In preparation for this new role, which will formally begin in April 2013, the Department of Health has asked NICE to run a pilot programme for developing social care quality standards using two topics. The two pilot topics referred to NICE are:

1. Care of people with dementia
2. Health and wellbeing of looked-after children and young people

NICE will develop these quality standards in collaboration with two topic expert groups; these independent groups are responsible for prioritising the areas that should be reflected in the quality standards.

The pilot will test NICE’s draft methods and processes, explore the format and presentation that the quality standards could take in social care settings and develop an approach to integrating these with NICE’s quality standards on healthcare. The consultation on both draft quality standards with stakeholders is expected to open in August and the final quality standards will be published in April 2013. Over time it is expected that a library of social care standards will be developed for priority topic areas.

## Conclusion

The Health and Social Care Act (2012) places NICE at the heart of the government’s plans to improve health and social care. Despite there being a very different evidence base for good practice in social care compared with healthcare, it is widely acknowledged that integrating the two is fundamental to achieving excellence in care. NICE is well qualified to take up this new remit and looks forward to building on its strong track record in developing evidence-based guidance in healthcare and public health.

NICE will formally cease to be a special health authority of the NHS next April and will be re-established as the National Institute for Health and Care Excellence but its core mission to help professionals to deliver the best possible care based on the best available evidence will continue.

To keep up to date with the latest developments about NICE’s new role in social care:

- Visit NICE’s website: www.nice.org.uk/socialcare
- Follow NICE on Twitter: www.twitter.com/NICEComms
- Subscribe to receive NICE’s newsletters and alerts: www.nice.org.uk/newsletter

